# The Probiotics in Pediatric Asthma Management (PROPAM) Study in the Primary Care Setting: A Randomized, Controlled, Double-Blind Trial with *Ligilactobacillus salivarius* LS01 (DSM 22775) and *Bifidobacterium breve* B632 (DSM 24706)

**DOI:** 10.1155/2022/3837418

**Published:** 2022-01-17

**Authors:** Lorenzo Drago, Luigi Cioffi, Maria Giuliano, Marco Pane, Angela Amoruso, Irene Schiavetti, Gregor Reid, Giorgio Ciprandi, PROPAM Study Group

**Affiliations:** ^1^Department of Biomedical Sciences for Health, University of Milan, Milan, Italy; ^2^Pediatric Primary Care ASL Napoli 2, Naples, Italy; ^3^Research and Development, Probiotical Research, Novara, Italy; ^4^Health Science Department, University of Genoa, Genoa, Italy; ^5^Departments of Microbiology, Immunology and Surgery, Western University, London, ON, Canada; ^6^Allergy Clinic, Casa di Cura Villa Montallegro, Genoa, Italy

## Abstract

**Background:**

Type-2 inflammation commonly marks asthma in childhood. Also, gut and lung dysbiosis is detectable in patients with asthma. Strain-related probiotic supplementation may restore a physiological immune response, dampen airway inflammation, and repair dysbiosis. Therefore, the probiotics in pediatric asthma management (PROPAM) study is aimed at demonstrating that *Ligilactobacillus salivarius* LS01 (DSM 22775) and *Bifidobacterium breve* B632 (DSM 24706) mixture could reduce asthma exacerbations in children, followed in a primary care setting.

**Methods:**

The study was randomized, placebo-controlled, and double-blind. It involved 11 Italian primary care pediatricians. The probiotic mixture (containing *Ligilactobacillus salivarius* LS01 1 × 10^9^ live cells and *Bifidobacterium breve* B632 1 × 10^9^ live cells) or placebo was taken twice daily (1 sachet in the morning and 1 in the evening) for eight weeks and subsequently once daily for a further eight weeks. Outcomes included number, severity, and duration of asthma exacerbations, intensity of maintenance and as need treatments, and safety.

**Results:**

The per-protocol population included 422 children (mean age seven years, 240 males and 182 females). The probiotic mixture significantly reduced the number of asthmatic exacerbations (OR = 3.17). In addition, the number of children with two exacerbations was less than a third in the active group (OR = 3.65).

**Conclusions:**

This PROPAM study demonstrated that probiotic strains *Ligilactobacillus salivarius* LS01 (DSM 22775) and *Bifidobacterium breve* B632 (DSM 24706) were safe and significantly reduced by more than a third the frequency of asthma exacerbations. At present, the first-line treatment of asthma is still drug-based, but specific strains of probiotics may be auxiliary remedies.

## 1. Introduction

Asthma, including wheezing, represents a severe global health problem and a relevant burden for the healthcare system, as underscored by the 2021 Global Initiative for Asthma (GINA) guidelines (www.ginasthma.org). Children with asthma usually have a type-2 phenotype and consequently are prone to have frequent respiratory infections [1]. Moreover, asthma exacerbations often follow acute respiratory infections in childhood [2]. As a result, modulation of the immune response and prevention of respiratory infections assume an essential role in the therapeutical strategy.

The increase in asthma prevalence has been initially attributed to the hygiene hypothesis, especially to the imbalance of human microbiota composition, abundance, and diversity (dysbiosis) that promotes the maintenance of type-2 phenotype in the infant [3]. This gut microbiota dysbiosis appears to play a role in increasing allergy prevalence [4]. It has been proposed that a decline in biodiversity determines microbial deprivation affecting the immune response. Indeed, children with asthma display lung and intestinal dysbiosis [5] likely promote the activation of inflammatory pathways and contribute to bronchial obstruction and airway hyperresponsiveness. Thus, dysbiosis and reduced microbial diversity dysregulate the bidirectional crosstalk across the gut-lung axis [6]. This axis provides a rational for understanding how oral supplements could improve respiratory illness [7]. These concepts have paved the way for manipulating the immune system using nonpharmacological remedies including probiotics.

There is a body of pathophysiological evidence supporting probiotic use in allergy and asthma [8]. Certain strains can promote the expansion of type-1 response, downregulate IgE production, and reinforce the immune defense against respiratory infections [9]. Studies have explored the potential effects of probiotics in preventing allergic diseases and asthma; however, the outcomes were conflicting because of a high degree of heterogeneity among studies, mainly concerning study design, populations, timing, considered variables, and overall used strains [10–13]. It is essential to consider the properties of strains including their genetic, adaptative, immunological, and metabolic characteristics, to better target the desired efficacy and safety. In the present study, the mixture of two well-characterized strains, *Bifidobacterium breve* B632 and *Ligilactobacillus salivarius* LS01, was based on microbiological and clinical evidence that supported their potential role in asthma management [14–17]. As a result, the PRObiotics in Pediatric Asthma Management (PROPAM) study tested the hypothesis that these strains could prevent asthma exacerbations in a pediatric primary care setting. The aim was, therefore, to evaluate the possible reduction of asthma exacerbations and improvement of disease severity.

## 2. Materials and Methods

The PROPAM study was designed as a randomized, placebo-controlled, and double-blind trial. The primary outcome was the reduction of asthma exacerbations, considering the number, duration (days), and severity of asthma attacks. The severity was assessed using the scoring provided by the Italian Society of Pediatrics [18]. It was graded as mild, moderate, and severe.

The secondary outcome was the reduction of drugs used in maintenance and as needed for asthma exacerbations.

The variables included (i) the number of asthma exacerbations, severity, and duration documented by parents in a diary and assessed by clinicians at visits; (ii) the maintenance therapy, assessed by the intensity of treatment, scored as follows: no treatment, low intensity (antileukotrienes alone), medium intensity (inhaled corticosteroids alone), high intensity (inhaled corticosteroids combined with long-acting *β*2-agonists and/or antileukotrienes), and (iii) the as-needed therapy during exacerbations, considering the use of oral corticosteroid and/or increase of inhaled corticosteroid dosage.

The eligibility criteria consisted of inclusion and exclusion criteria. The inclusion criteria were age between 3 and 14 years, and asthma diagnosis, according to GINA criteria. The exclusion criteria were severe asthma, congenital or acquired immunodeficiency, cystic fibrosis, and chronic pulmonary diseases.

The study started on April 2017 and finished in November 2019. The treatment period lasted 16 weeks.

The study included five visits: at baseline (T0), after one month of treatment (T1), two months (T2), three months (T3), and four months (T4). During each visit, the investigator performed detailed medical history, mainly concerning asthma exacerbations and use of medications, physical examination, drug countability, and revised the therapeutic strategy if necessary.

Each sachet of the active probiotic product comprised viable strains currently used in food supplements, specifically ≥1 × 10^9^ live cells of *B. breve* B632 (DSM 24706) and ≥1 × 10^9^ live cells of *L. salivarius* LS01 (DSM 22775) (combined dose of ≥2 × 10^9^ live), with maltodextrin used as a bulking agent to yield a final weight of 2 grams; each placebo sachet contained 2 grams of maltodextrin only (Probiotical S.p.A., Novara, Italy). The placebo powder was indistinguishable from the probiotic powder in appearance, taste, smell, and packaging. Participants were instructed to dissolve the powder in water or cold milk and drink it in the morning and evening. The probiotic sachets were analyzed by Biolab Research S.r.l., Novara, Italy, via flow cytometry (ISO 19344 : 2015 IDF 232 : 2015, ≥2 × 10^9^ active fluorescent units (AFU)) and plate count method (Biolab Research Method 014-06, ≥2 × 10^9^ CFU) to confirm target cell count.

The sample size was calculated to power the study to detect a 25% reduction of asthma episodes. This required enrollment of 200 subjects per arm. Assuming a drop-out rate of 20%, a total of 500 children enrolled was judged adequate to provide sufficient quantity to detect the stated percentage. The estimates were obtained, setting the probability of type I error *α* = 0.05 (two-tailed) and a CI width of 0.14. Participants were enrolled and randomized with a 1.1 ratio. The randomization method was computer-generated. Globally, 11 Italian primary care pediatricians participated in the study, each having to enroll 46 children. All of them resided in the Campania (South Italy) region.

The Ethics Committee of the ASL Napoli 3 Sud approved the study procedure on April 12, 2017 (N. 45/21/04/2017).

The statistical assessment included a descriptive analysis of collected data, summarized as counts within a group for categorical variables and with mean ± standard deviation and median with interquartile range for continuous variables. Univariate logistic regression models were applied to predict the outcomes' likelihood (presence of asthma exacerbation during the treatment period). Results were quantified by odds ratio (OR) together with a 95% confidence level (95% CI). The significance level was set at 0.05. The analyses were computed using SPSS Statistics version 21.0 (IBM Corp., Armonk, NY, USA).

## 3. Results

**Figure 1:**
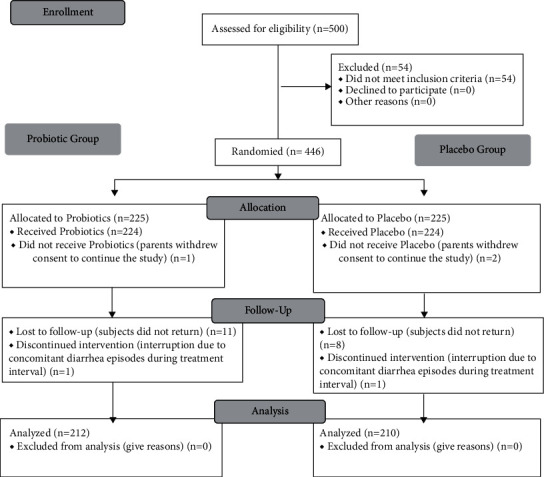
CONSORT flowchart.

The pediatricians enrolled 500 children who were screened for clinical trial eligibility.  Figure 1 reports the patient disposition. The analyzed per-protocol (PP) population consisted of 422 children: 212 in the active arm and 210 in the placebo one.

### 3.1. General Characteristics

Table 1 summarized the demographic and clinical data in the PP population at baseline (T0). It included 422 children (mean age 7 + 3.17 years), 182 females, and 240 males. Of these, 291 resided in the city and 131 in rural communities. One hundred and seventy-six subjects (41.7%) reported passive smoking. Only 50 (11.8%) did not go to school. Family atopy was declared for 320 (75.8%) children, and sensitization was reported in 210 (49.8%) children.

The analysis stratified the patient population into an active group and a placebo group. The intergroup comparison showed no significant differences between study groups in all demographic and clinical characteristics at baseline (Table 1). Thus, the two groups were well-matched.

### 3.2. Primary Outcomes

Fifty (23.8%) children in the placebo group experienced at least one asthma exacerbation compared to 19 (9%) in the active group (Table 2). Seventeen (8.1%) children of the placebo group and 5 (2.4%) in the active group had two asthma exacerbations. In total, there were 67 asthma exacerbations in the placebo group and 24 in the active group. The univariate logistic regression analysis showed that children in the placebo group had a higher probability of having at least one asthma exacerbation than children in the probiotic arm: (OR 3.17, 95% CI 1.8–5.6; *p* < 0.001). In addition, children in the placebo group were more likely to have two exacerbations than children in the active group (OR 3.65, 95% CI 1.32–10.08; *p* = 0.013). In other words, the probiotic mixture reduced nearly to a quarter the probability of having two asthma exacerbations.

In terms of severity of asthma exacerbations, children in the placebo group had 21 (31.3%) mild episodes, 44 (65.7%) moderate episodes, and 2 (3.0%) severe. In the active arm, 4 (16.7%) children had mild asthma exacerbations, 19 (79.2%) moderate, and 1 (4.1%) severe (Table 3). The mean asthma exacerbation duration was 3.3 + 2.57 days in the placebo group and 3.3 + 2.45 in the active arm (Table 3).

### 3.3. Secondary Outcomes

There was a reduction trend for treatment intensity over time (Table 4). No significant difference was observed at the intragroup and intergroup analyses, even if there were some missing data in the database. In particular, more than 40% of children did not take any treatment at *T*0. This high figure depended on the asthma severity: more than 80% of children had intermittent asthma. The frequency of children without treatment was inclined to increase in both groups over time.  Table 5 shows the use of oral and inhaled corticosteroids during asthma exacerbations. Oral corticosteroids were prescribed in about 70% of children, and inhaled corticosteroid dosage was increased in about 50% of them. So, no significant difference between groups was reported.

### 3.4. Safety Data

Both treatments were well-tolerated, and no clinically relevant adverse event was reported.

## 4. Discussion

The present study showed that the probiotic mixture containing *B. breve* B632 and *L. salivarius* LS01 significantly reduced the frequency and severity of asthmatic exacerbations in a primary care setting that accurately represents what occurs in daily practice.

Asthma exacerbations represent an important issue in pediatric clinical practice [19]. In particular, asthma exacerbation is closely associated with asthma severity requiring that children require effective management in primary care settings. In childhood, acute upper airway infection, mainly of viral origin, represents the leading cause of asthma relapse [20]. Viral infections implicate bronchial inflammation that triggers airway hyperresponsiveness and further narrows the bronchial lumen. Moreover, allergic subjects are more susceptible to frequent and severe infections than nonallergic subjects [21]. As a result, a vicious circle includes asthma, allergy, infections, and acute respiratory episodes. These phenomena depend on the overexpression of type-2 immune response usually involved in pediatric asthma. It is essential to adopt preventive measures to restore a physiological immune response [22]. The encouraging results here reflect such measures.

A dysbiosis can be generally defined as a reduction in microbial diversity and a combination of the loss of beneficial bacteria such as certain members of the Bacteroides or Firmicutes phyla and a rise in pathobionts (bacteria that become pathogenic under certain conditions), including Proteobacteria. This imbalance may lead to a dysregulated immunological response in several organs, including the lung [23]. Probiotic strains can modulate the immune system by restoring a physiologic type-1 response, dampening inflammation, and reactivating eubiosis [7]. Examples include a demonstrating the inefficacy of a one-year consumption of fermented milk with *Lactobacillus casei* in 187 asthmatic preschoolers [24], and another in which *Lactobacillus rhamnosus* GG use reduced episodes in infants with at least two wheezing episodes and family atopy [25].

Another study showed that eight-week *Lactobacillus gasseri* A5 supplementation in asthmatic children improved lung function, reduced asthma symptom scores, and increased asthma control test (ACT) score significantly diminishing the production of TNF-*α*, IFN-*γ*, IL-12, and IL-13 by peripheral blood mononuclear cells [26]. Some bifidobacteria have also shown benefits, with a combination of *Bifidobacterium longum* BB536, *Bifidobacterium infantis* M-63, and *Bifidobacterium breve* M-16V, being shown to significantly reduce respiratory symptoms and improve quality of life (QoL), unlike the placebo [27]. One strain, *Bifidobacterium lactis* Probio-M8, was found to provide added value when ingested along with inhaled Symbicort, by decreasing FeNO, improving ACT, and modulating gut biodiversity [28].

These outcomes are relevant to clinical practice. Asthma exacerbations, mainly if associated with hospitalization, create a burden for the healthcare system and negatively affect children and their families.

The present findings emphasize the importance of selecting strains, since both used here showed positive effects in other disease models [14–17]. The *L salivarius* strain also can produce tolerogenic peptides, restore the physiological type-1 polarization, expand T regulatory cells, and improve epithelial barrier function [7–9].

The study does have some limitations. It did not examine longer treatment nor specifically examine mechanistic parameters. In addition, there are many reasons for asthma exacerbation, including respiratory infections, allergen exposure, unauthorized drug withdrawal, exercise, meteorological change, and pollutants. The current study did not analyze the precise reasons for the subjects' asthma exacerbation and the mechanism by which probiotics reduce asthma exacerbations cannot be speculated. Moreover, there were some missing data for some variables, mainly concerning the treatment details. It could happen in studies conducted in a primary care setting.

Nevertheless, the finding that probiotic strains, readily available for consumers, can reduce asthma exacerbations in children provides a potential complementary therapy for primary care physicians.

## 5. Conclusions

The PROPAM study provided clinical evidence suggesting that *Bifidobacterium breve* B632 (DSM 24706) and *Ligilactobacillus salivarius* LS01 (DSM 22775) may prevent asthma exacerbations in children. Moreover, this probiotic mixture was safe and well-tolerated. At present, the first-line treatment of asthma is still drug-based, but specific strains of probiotics may be auxiliary remedies.

## Figures and Tables

**Table 1:** Baseline characteristics.

	Total *N* = 422	Placebo *N* = 210	Active treatment *N* = 212
Age	7.0 ± 3.17	7.0 ± 2.95	7.0 ± 3.38
Sex			
Female	182 (43.1%)	91 (43.3%)	91 (42.9%)
Male	240 (56.9%)	119 (56.7%)	121 (57.1%)
Living			
City	291 (69.0%)	143 (68.1%)	148 (69.8%)
Rural	131 (31%)	67 (31.9%)	64 (30.2%)
Passive smoking at home			
No	246 (58.3%)	125 (59.5%)	121 (57.1%)
Father	76 (18.0%)	39 (18.6%)	37 (17.5%)
Mother	32 (7.6%)	15 (7.1%)	17 (8.0%)
Both	57 (13.5%)	26 (12.4%)	31 (14.6%)
Other	11 (2.6%)	5 (2.4%)	6 (2.8%)
School attendance			
No	50 (11.8%)	24 (11.4%)	26 (12.3%)
Yes	372 (88.2%)	186 (88.6%)	186 (87.7%)
Family atopy			
Yes	320 (75.8%)	168 (80.0%)	152 (71.7%)
No	75 (17.8%)	30 (14.3%)	45 (21.2%)
Sensitized children			
Yes	210 (49.8%)	104 (49.5%)	106 (50%)
No	212 (50.2%)	106 (50.5%)	106 (50%)

**Table 2:** Number and frequency of children with or without asthma exacerbations during the study.

	Placebo	Active treatment	OR (95%IC); *p*
No exacerbation	160 (76.2%)	193 (91.0%)	3.17 (1.80–5.60); <0.001
At least one exacerbation	50 (23.8%)	19 (9.0%)	
Less than two exacerbations	193 (91.9%)	207 (97.6%)	3.65 (1.32–10.08); 0.013
Two exacerbations	17 (8.1%)	5 (2.4%)	

**Table 3:** Severity and duration of exacerbations during the study.

	Placebo	Active treatment
Total number of exacerbations	67	24
Severity		
Mild	21 (31.3%)	4 (16.7%)
Moderate	44 (65.7%)	19 (79.2%)
Severe	2 (3.0%)	1 (4.2%)
Duration (days)	3.3 ± 2.57	3.3 ± 2.45

**Table 4:** Maintenance therapy over time: intensity of asthma treatment in both groups.

	Placebo				Active treatment			
	No therapy	Low intensity	Mild intensity	High intensity	No therapy	Low intensity	Mild intensity	High intensity
*T*0	83 (40.5%)	40 (19.5%)	38 (18.5%)	44 (21.5%)	95 (45.5%)	36 (17.2%)	30 (14.4%)	48 (23.0%)
*T*1	86 (42.2%)	41 (20.1%)	37 (18.1%)	40 (19.6%)	95 (46.6%)	33 (16.2%)	28 (13.7%)	48 (23.5%)
*T*2	99 (49.0%)	35 (17.3%)	29 (14.4%)	39 (19.3%)	98 (48.3%)	33 (16.3%)	31 (15.3%)	41 (20.2%)
*T*3	103 (51.5%)	33 (16.5%)	27 (13.5%)	37 (18.5%)	104 (51.7%)	38 (18.9%)	29 (14.4%)	30 (14.9%)
*T*4	123 (61.8%)	23 (11.6%)	25 (12.6%)	28 (14.1%)	115 (57.2%)	32 (15.9%)	24 (11.9%)	30 (14.9%)

**Table 5:** Treatment during asthma exacerbations in both groups.

	Placebo (67 exacerbations)	Active treatment (24 exacerbations)
Use of oral corticosteroids	45 (67.2%)	17 (70.8%)
Increased dosage of inhaled corticosteroids	34 (50.7%)	13 (54.2%)

## Data Availability

The clinical data used to support the findings of this study are available from the corresponding author upon request.

## Additional Points

*Key Message.* Specific strains (*Ligilactobacillus salivarius* LS01 (DSM 22775) and *Bifidobacterium breve* B632 (DSM 24706)) may prevent asthma exacerbations in children.
